# Brain microstructure mediates sex-specific patterns of cognitive aging

**DOI:** 10.18632/aging.202561

**Published:** 2021-01-28

**Authors:** Emilie T. Reas, Donald J. Hagler, Allison J. Zhong, Roland R. Lee, Anders M. Dale, Linda K. McEvoy

**Affiliations:** 1 Department of Neurosciences, University of California, San Diego, La Jolla, CA 92093, USA; 2 Department of Radiology, University of California, San Diego, La Jolla, CA 92093, USA; 3 New York Medical College, Valhalla, NY 10595, USA; 4 Radiology Services, VA San Diego Healthcare System, La Jolla, CA 92093, USA; 5 Department of Family Medicine and Public Health, University of California, San Diego, La Jolla, CA 92093, USA

**Keywords:** diffusion MRI, cognitive function, normal aging, memory, sex differences

## Abstract

Normal brain aging is characterized by declining neuronal integrity, yet it remains unclear how microstructural injury influences cognitive aging and whether such mechanisms differ between sexes. Using restriction spectrum imaging (RSI), we examined sex differences in associations between brain microstructure and cognitive function in 147 community-dwelling older men and women (56-99 years). Gray and white matter microstructure correlated with global cognition, executive function, visuospatial memory, episodic memory, and logical memory, with the strongest associations for restricted, hindered and free isotropic diffusion. Associations were stronger for women than for men, a difference likely due to greater age-related variability in cognitive scores and microstructure in women. Isotropic diffusion mediated effects of age on cognition for both sexes, though distinct mediation patterns were present for women and men. For women, hippocampal and corpus callosum microstructure mediated age effects on verbal and visuospatial memory, respectively, whereas for men fiber microstructure (mainly fornix and corpus callosum) mediated age effects on executive function and visuospatial memory. These findings implicate sex-specific pathways by which changing brain cytoarchitecture contributes to cognitive aging, and suggest that RSI may be useful for evaluating risk for cognitive decline or monitoring efficacy of interventions to preserve brain health in later life.

## INTRODUCTION

Even in the absence of dementia, advancing age is accompanied by variable rates of cognitive decline, posing substantial challenges to defining normal cognitive aging and identifying its neurobiological substrates. While cognitive decline may be an inherent consequence of dysfunctional senescent cells, environmental and lifestyle factors may compound basal rates of cell damage, leading trajectories of neuroanatomical change to diverge from those expected for chronological age. Postmortem studies have demonstrated characteristic cytoarchitectural features of the aged brain, including lower dendritic and axonal density, demyelination, and reduced dendritic branching [1]. However, histological studies capable of visualizing cellular morphometry cannot capture changes in the healthy living brain or link structural changes with functional outcomes. Leveraging advanced *in vivo* imaging tools to identify patterns of cytoarchitectural injury that underlie cognitive decline will enhance our understanding of typical brain aging, ultimately helping to disentangle markers of normal from pathological cognitive deficits, and to guide interventions to preserve cognitive health through the end of life.

Fluid cognitive functions, notably in the executive and memory domains, are most vulnerable to age-related decline, with relative sparing of crystallized knowledge [2, 3]. Diffusion tensor imaging (DTI) studies, which allow inference of tissue microstructure from the magnitude and orientation of water diffusion, have identified regionally non-specific decreases in fiber fractional anisotropy (FA) and increases in mean diffusivity (MD) that correspond with age-related decline in these cognitive functions [4–8]. Such findings have prompted theories that cortical disconnection drives cognitive aging [9], yet whether neuroanatomical changes lie along a causal pathway from aging to cognitive decline has been debated [10, 11]. DTI studies have offered evidence that white matter microstructure mediates age-related variability in visuospatial function, reasoning, executive function, and perceptual speed, but not in memory, verbal fluency or global cognitive function [12–17]. However, conventional white matter DTI measures offer an obscured window onto the underlying cellular microarchitecture and their associated cortical changes.

Diffusion models that expand upon the single tensor model enable finer characterization of tissue microstructure by dissociating diffusion among distinct cellular compartments and resolving within-voxel structural complexities. Studies using neurite orientation and dispersion density imaging (NODDI) have reported that reduced frontal white matter neurite density correlates with learning and executive function in late-middle age [18], and that orientation dispersion in hippocampal and frontal cortex mediates correlations between age and executive function across the adult lifespan [19]. However, it is unclear the extent to which cognitive deficits emerging with age are attributable to cytoarchitectural injury, or how factors besides age promote microstructural damage that contributes to functional impairments.

Sex has been shown to moderate risk for cognitive impairment in late life, with women exhibiting heightened risk for dementia and more pronounced clinical and cognitive manifestations of pathology and risk factors [20]. Yet it is undetermined whether trajectories of typical brain aging similarly differ by sex. More rapid atrophy has been observed for both women [21] and men [22]. Sex differences in microstructural change with age have also been inconclusive [23–25] and have been inadequately examined relative to cognitive outcomes.

Restriction spectrum imaging (RSI) models diffusion orientation along a spectrum of length scales from multi-shell, multi-orientation diffusion MRI, allowing separation of restricted (intraneurite), hindered (extraneurite) and free water (CSF) compartments [26]. We recently observed cortical and white matter increases in free water and decreases in restricted and hindered diffusion with age in community-dwelling older adults, with stronger correlations for women than men [27]. We previously found that reduced restricted diffusion and increased free water predicted cognitive decline in mild cognitive impairment [28, 29], though these measures have not been examined in the context of more subtle cognitive declines that occur with typical aging.

In this study of community-dwelling older adults we expand our findings of age-microstructure correlations to examine associations of gray and white matter RSI metrics with performance in cognitive domains known to decline in later life. We assessed whether associations between microstructure and cognitive function differed in strength or topography between men and women, and whether such differences were driven by our previously observed sex-dependent effects of age on brain microstructure. We further investigated whether microstructure mediates effects of age on cognitive function.

## RESULTS

### Participant characteristics

One-hundred forty-seven participants of the Rancho Bernardo Study (RBS) of Healthy Aging completed RSI scanning and a neuropsychological battery including measures of global cognition (Modified Mini Mental Status Examination; 3MS), executive function (Trail Making Test, Part B; Trails B), verbal episodic memory (Buschke Selective Reminding Test, total and delayed recall), visuospatial memory (Wechsler Visual Reproduction test, immediate and delayed recall), and logical memory (Wechsler Logical Memory subtest, immediate and delayed recall). Participant characteristics, cognitive test scores, and global RSI measures (the mean across all cortical gray matter or all white matter fibers) by sex are shown in Table 1. Participants were 61% women and had a mean age of 76.6±7.8 years (range 56-99). Women had less education (*p*<0.001), lower body mass index (BMI, kg/m^2^) (*p*=0.003) and rates of hypertension (*p*=0.04), and were more likely to be unmarried or live alone (*p*<0.001) than men. Women scored higher on the Buschke total (*p*=0.004) and delayed (*p*=0.02) recall tests. Older age strongly correlated (*r*>0.50, adjusted for education) with worse performance on all cognitive tests except logical memory for women and more weakly correlated with cognitive scores for men (*r*<0.50) (Supplementary Table 1), with significantly stronger correlations between age and Buschke recall for women than for men.

**Table 1:** Participant characteristics, cognitive test scores, and mean RSI measures by sex (mean±SD).

	**Women (N=90)**	**Men (N=57)**
Age (years)	76.2±7.8	77.3±7.9
Education (years)	14.4±1.9	15.8±2.0 ***
Exercise (% 3+times/week)	74	79
Marital status (% married)	66	95 ***
Living status (% cohabitating)	72	95 ***
Smoking (% ever)	37	47
Alcohol consumption (% drinker)	87	82
BMI	25.2±4.3	27.1±3.1 **
Hypertension (%)	53	70 *
Diabetes (%)	8	16
3MS	94.8 ± 4.6	94.9 ± 4.8
Trails B	87 ± 44	89 ± 38
BTR	40.2 ± 8.0	36.1 ± 7.9 **
BDR	6.9 ± 2.4	6.0 ± 2.3 *
VRI	12.3 ± 4.3	12.9 ± 3.9
VRD	8.6 ± 5.2	9.4 ± 5.1
LMI	13.2 ± 3.9	12.3 ± 3.9
LMD	11.9 ± 4.6	10.5 ± 4.5
Fiber RI	0.42±0.03	0.42±0.02
Fiber ND	0.60±0.02	0.60±0.02
Fiber IF	0.25±0.03	0.26±0.03
Gray matter RI	0.29±0.02	0.29±0.02
Gray matter ND	0.22±0.01	0.22±0.01
Gray matter HI	0.74±0.04	0.72±0.04 **
Gray matter IF	0.41±0.06	0.44±0.06 **

* *p*<0.05, ** *p*<0.01, *** *p*<0.001.

Cognitive test scores are adjusted for education.

BTR, Buschke total recall; BDR, Buschke delayed recall; HI, hindered isotropic; IF, isotropic free water; LMI, logical memory immediate recall; LMD, logical memory delayed recall; ND, neurite density; RI, restricted isotropic; VRI, visual reproduction immediate recall; VRD, visual reproduction delayed recall.

### Associations of global microstructure with sex and age

RSI metrics of interest were computed at the whole brain level (across all white matter fibers and across all cortical gray matter) and included restricted isotropic (RI), neurite density (ND), hindered isotropic (HI), and isotropic free water (IF) diffusion. Restricted measures (RI, ND) are presumed to reflect diffusion within the intracellular compartment (cell bodies and neurites, respectively), HI is presumed to reflect diffusion within large cell bodies or in the extracellular space, and IF is presumed to reflect CSF. Results showed that women had higher global gray matter HI and lower gray matter IF than men (Table 1; *p*<0.01), but restricted RSI measures did not differ by sex (*p*>0.05). Older age correlated with lower global RI, ND and HI, and with higher global IF, with generally stronger correlations for women (most *r*s>0.60) than men (most *r*s>0.30) (Supplementary Table 1). Correlations with age were significantly stronger for women than men for global fiber RI and ND, and for global gray matter IF and HI (*p*<0.01, Fisher r-to-z transformation).

### Associations between global microstructure and cognitive function

Correlations between cognitive function and brain microstructure at the whole-brain level were computed for men and women separately (adjusted for education) and correlation strengths were compared between sexes (Fisher’s r-to-z transformation). One or more global white and gray matter microstructure metrics demonstrated strong correlations (*r*>0.50) with scores on all cognitive tests except logical memory for women, and with visual recall for men (Supplementary Table 1). Better performance correlated with higher RI, ND and HI, and lower IF. Correlations were significantly stronger for women than for men between Buschke recall and fiber RI, ND, and IF (*p*<0.05).

Because social factors including marital status or residential situation may affect brain or cognitive health, sensitivity analyses were conducted with additional adjustment for marital status (married/unmarried) and living status (alone/cohabitating). For women, correlation strengths were marginally attenuated, but the pattern of associations was unchanged (Supplementary Table 2). Strong correlations (*r*>0.50) remained for visual recall, and moderate correlations (*r*>0.30) remained for all other cognitive measures with gray and white matter microstructure. Due to an insufficient number of men who were unmarried or living alone (*N*=3), adjustment for marital and living status was not performed among men.

### Associations between regional cortical gray matter microstructure and cognitive function

**Figure 1:**
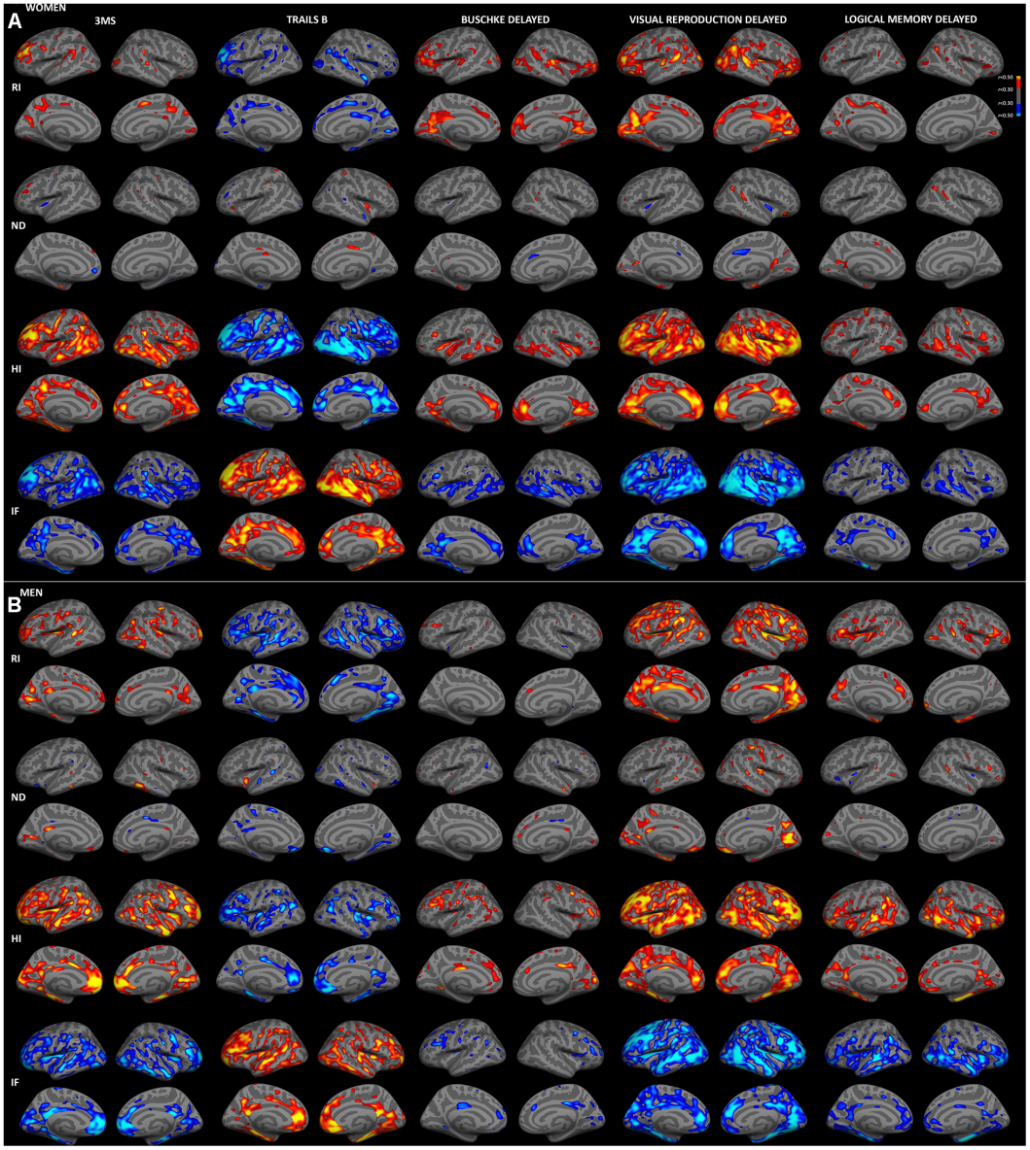
**Sex-stratified associations between cortical gray matter microstructure and cognitive function.** Partial correlations between RSI metrics and cognitive test scores, adjusted for education, are shown for women (**A**) and men (**B**). Warm colors indicate positive correlations and cool colors indicate negative correlations (*r*>0.30). For Trails B, lower scores indicate better performance. (HI, hindered isotropic; IF, isotropic free water; ND, neurite density; RI, restricted isotropic).

To examine topographic associations between cortical gray matter microstructure and cognitive function, vertex-wise regressions were computed for men and women (adjusted for education), as illustrated in Figure 1. Results for immediate recall (Supplementary Figure 1) were comparable to those for delayed recall. Generally, associations with RSI were stronger for 3MS, Trails B, and visual recall than for Buschke recall and logical memory. For both sexes, moderate correlations (*r*>0.30) were present between better cognitive performance and higher RI and HI and lower IF. There were minimal associations between cognitive function and ND, with the exception of correlations between better visual recall and higher ND for men.

**Figure 2:**
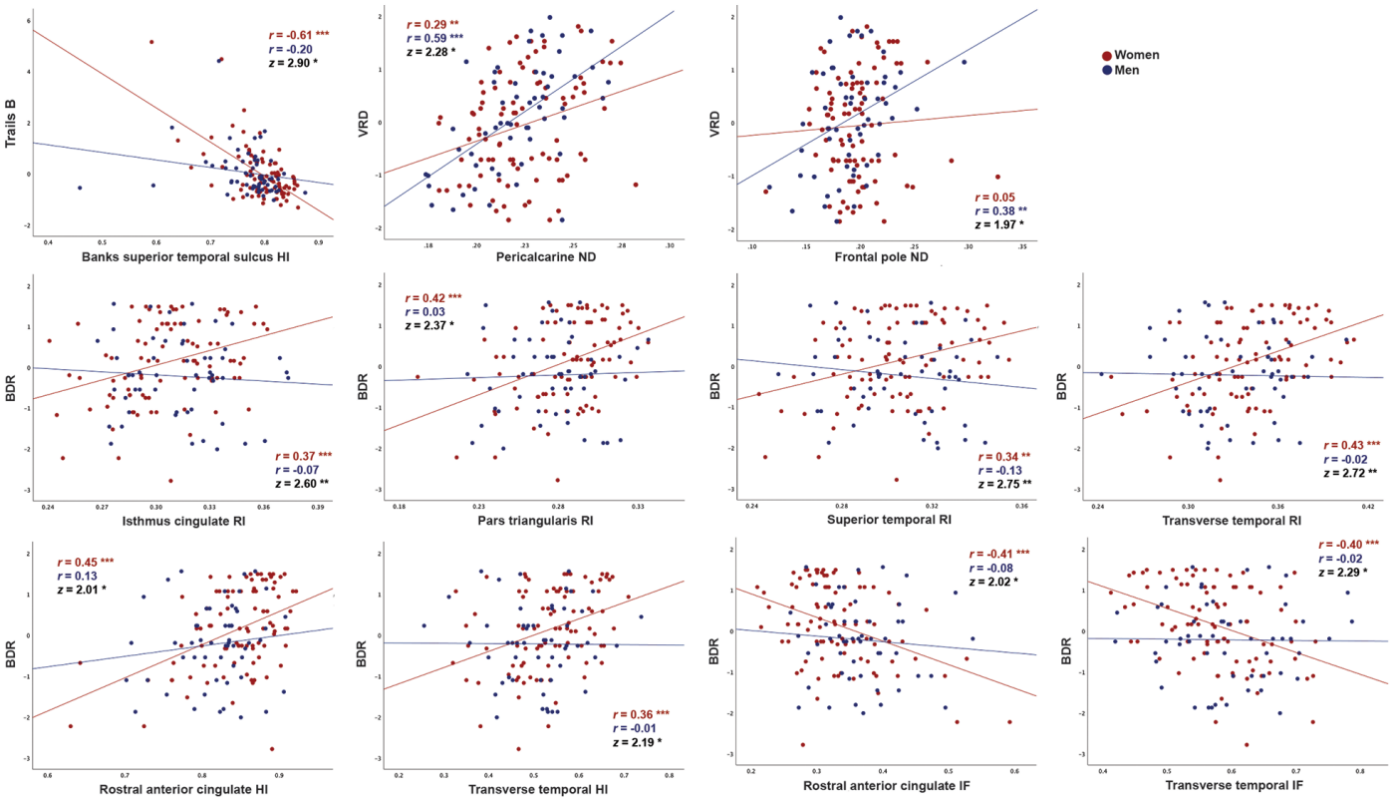
**Correlations between cortical gray matter microstructure and cognitive function in regions demonstrating significant differences in correlation strength between men and women.** Correlations between RSI metrics and cognitive test scores (residuals after adjustment for education using linear regression), are shown for women and men. Data are shown only for regions in which correlations were moderate for either women or men (*r*>0.30) and differed in strength between sexes (*p*<0.05, Fisher r-to-z transformation). For Trails B, lower scores indicate better performance. (BDR, Buschke delayed recall; HI, hindered isotropic; IF, isotropic free water; ND, neurite density; VRD, visual reproduction delayed recall) * *p*<0.05, ** *p*<0.01, *** *p*<0.001.

Consistent with whole-brain associations, distinct patterns of correlations between regional microstructure and cognitive scores were observed for men and women. When correlation strengths were compared across cortical surface vertices, several small clusters demonstrated significant sex differences (Fisher r-to-z transformation, *p*<0.01, uncorrected; Supplementary Figure 2), although no clusters survived FDR correction. Most notably, stronger correlations were observed for women between Trails B scores and superior and middle temporal HI, and for men between visual recall scores and right medial occipital ND. Comparisons were also conducted within ROIs demonstrating qualitative sex differences on the surface maps illustrated in Figure 1. As shown in Figure 2, within these ROIs correlations were stronger (*p*<0.05) for women between Trails B scores and HI in the superior temporal sulcus, between Buschke recall and RI in isthmus cingulate, pars triangularis, and superior and transverse temporal cortices, and for Buschke recall with HI and IF in rostral anterior cingulate and transverse temporal cortex. Men showed stronger correlations between visual recall and ND in pericalcarine sulcus and frontal pole.

### Comparison between RSI-cognition and thickness-cognition associations

Sex-stratified correlations between cognitive function and cortical thickness, adjusted for education, are shown in Supplementary Figure 3. Comparison with Figure 1 suggests partial topographic overlap between RSI-cognition and thickness-cognition correlations, particularly for isotropic metrics (RI, HI, IF). When correlations between RSI metrics and cognitive measures were additionally adjusted for vertex-wise cortical thickness, strong correlations persisted (Supplementary Figure 4), suggesting that associations between microstructure and cognition are not attributable to atrophy.

### Associations of hippocampal and fiber microstructure with cognitive function

Sex-stratified correlations of hippocampal and fiber tract microstructure with cognitive function, adjusted for education, are shown in Table 2 (correlations for immediate recall measures are shown in Supplementary Table 3). Similar to cortical effects, correlations for fiber tracts were marginally stronger for isotropic diffusion (RI and IF) than for ND. Strong correlations (*r*>0.50) were present for Trails B, Buschke recall and visual recall with microstructure in fornix, anterior thalamic radiation, and several association and commissural fibers. Comparable to sex differences in cortical gray matter, correlations with microstructure were stronger for women than for men for Buschke recall in the hippocampus and in most fiber tracts, with the most consistent sex differences for RI and IF (*p*<0.05). In addition, 3MS scores more strongly correlated with uncinate IF for women, and logical memory delayed recall more strongly correlated with cingulum IF for men (*p*<0.05).

**Table 2:** Partial correlations (partial r), adjusted for education, between hippocampal and fiber tract RSI metrics and cognitive test scores, stratified by sex.

		**Women**					**Men**				
**Region**	**Measure**	**3MS**	**Trails B**	**BDR**	**VRD**	**LMD**	**3MS**	**Trails B**	**BDR**	**VRD**	**LMD**
Hippocampus	RI	0.06	-0.09	0.29	0.31	0.12	0.06	-0.29	0.02	0.24	0.26
	ND	0.13	-0.10	0.34 †	0.43	0.22	0.02	-0.30	-0.03	0.31	0.25
	IF	-0.49	0.34	-0.52 * †	-0.34	-0.35	-0.38 *	0.24	-0.18	-0.21	-0.48 *
	HI	0.43 *	-0.35	0.35	0.14	0.29	0.35 *	-0.04	0.19	0.12	0.27
Fornix	RI	0.32	-0.41	**0.51** †	**0.50**	0.26	0.32	-0.53 *	0.04	0.56 *	0.42
	ND	0.23	-0.37	0.40 †	**0.51**	0.27	0.35	-0.38	-0.01	0.43	0.36
	IF	-0.37	0.49	**-0.50** †	**-0.53**	-0.32	-0.37 *	0.40	-0.12	-0.59 *	-0.45
Cingulum	RI	0.23	-0.33	0.38 †	0.47	0.24	0.15	-0.42 *	-0.03	0.41	0.32
	ND	0.04	0.05	0.14	0.07	0.19	0.20	-0.20	-0.06	0.15	0.02
	IF	-0.23	0.14	-0.12	-0.17	-0.06	-0.12	0.34	-0.12	-0.46 *	-0.48 * †
Parahippocampal	RI	0.12	-0.16	0.24 †	0.32	0.13	0.03	-0.34	-0.11	0.24	0.12
Cingulum	ND	0.10	-0.04	0.18	0.28 †	0.14	-0.07	-0.01	-0.24 †	-0.10	0.05
	IF	-0.33	0.18	-0.12	-0.14	-0.04	-0.22	0.19	-0.02	-0.40 *	-0.33
CST	RI	0.19	-0.33	0.44 †	0.42	0.31	0.33	-0.37	0.07	0.28	0.17
	ND	0.12	-0.22	0.05	0.27	0.17	0.06	-0.22	-0.19	0.11	0.17
	IF	-0.29	0.40	-0.31 †	-0.51	-0.32	-0.21	0.34	0.08	-0.32	-0.28
ATR	RI	0.30	-0.41	**0.50** †	**0.56**	0.28	0.18	-0.41	-0.01	0.42	0.35
	ND	0.33	-0.32	0.42 †	**0.51**	0.27	0.25	-0.38	-0.02	0.33	0.24
	IF	-0.43	0.47	**-0.55** †	**-0.59**	-0.38	-0.39 *	0.46	-0.17	-0.51 *	-0.41
Uncinate	RI	0.22	-0.36	0.34 †	0.44	0.22	0.03	-0.35	-0.12	0.26	0.27
	ND	0.15	-0.23	0.23	0.34	0.26	0.11	-0.16	-0.10	0.05	0.03
	IF	-0.49 †	0.44	-0.47 †	-0.46	-0.35	-0.17	0.27	-0.02	-0.30	-0.38
ILF	RI	0.10	-0.26	0.27	0.36	0.20	0.10	-0.38	-0.02	0.41	0.27
	ND	0.27	-0.21	0.32	0.35	0.23	0.28	-0.26	-0.05	0.21	0.16
	IF	-0.42	0.47	-0.41 †	-0.45	-0.25	-0.30	0.36	-0.03	-0.52 *	-0.43 *
IFO	RI	0.21	-0.36	0.39 †	0.46	0.26	0.13	-0.38	-0.09	0.34	0.27
	ND	0.29	-0.30	0.35 †	0.44	0.32	0.26	-0.20	-0.08	0.22	0.24
	IF	-0.38	**0.51**	-0.45 †	**-0.54**	-0.28	-0.31	0.33	-0.06	-0.49 *	-0.42
Forceps major	RI	0.24	-0.31	0.37 †	0.43	0.22	0.16	-0.36	-0.09	0.28	0.18
	ND	0.31	-0.26	0.32	0.42	0.24	0.31	-0.39 *	0.20	0.49 *	0.26
	IF	-0.39	0.39	-0.42 †	**-0.56**	-0.25	-0.37 *	0.46 *	-0.10	-0.52 *	-0.38
Forceps minor	RI	0.34	-0.36	0.46 †	**0.50**	0.33	0.20	-0.39	-0.13	0.25	0.29
	ND	0.38	-0.44	0.44 †	0.57 * †	0.34	0.23	-0.41 *	-0.16	0.23	0.23
	IF	-0.48	0.34	-0.48 †	**-0.50**	-0.36	-0.31	0.41 *	0.06	-0.34	-0.35
CC	RI	0.37	-0.45	0.47 †	**0.55**	0.36	0.28	-0.45 *	-0.04	0.39	0.25
	ND	0.39	-0.43	0.43 †	0.58 *	0.33	0.38	-0.44 *	0.01	0.40	0.25
	IF	-0.47	0.44	-0.49 †	-0.63 *	-0.34	-0.44 *	0.50 *	-0.05	-0.55 *	-0.33
SLF	RI	0.20	-0.30	0.36	0.34	0.27	0.37 *	-0.35	0.09	0.35	0.33
	ND	0.19	-0.06	0.19	0.19	0.32	0.20	-0.23	-0.10	0.14	0.15
	IF	-0.30	0.26	-0.29	-0.37	-0.26	-0.18	0.31	-0.15	-0.39	-0.37
SCS	RI	0.27	-0.39	0.45 †	0.48	0.33	0.42 *	-0.44 *	0.09	0.36	0.28
	ND	0.03	-0.15	0.05	0.22	0.14	-0.06	-0.01	-0.19	0.07	0.03
	IF	-0.30	0.40	-0.37 †	**-0.50**	-0.31	-0.10	0.25	0.02	-0.29	-0.30
SIFC	RI	0.34	-0.47	0.47 †	**0.56**	0.34	0.23	-0.43	-0.02	0.40	0.41
	ND	0.21	-0.26	0.20	0.31 †	0.29	-0.04	-0.08	-0.12	-0.14	0.02
	IF	-0.41	0.45	-0.42 †	**-0.53**	-0.35	-0.24	0.30	-0.01	-0.32	-0.38
IFSFC	RI	0.29	-0.40	0.41 †	0.43	0.34	0.35 *	-0.47 *	0.04	0.37	0.30
	ND	0.25	-0.35	0.24 †	0.40	0.30	0.07	-0.31	-0.16	0.18	0.17
	IF	-0.42	0.43	-0.33	-0.48	-0.30	-0.18	0.37	-0.03	-0.40 *	-0.31

Bold values indicate *r*>0.50 (*p*<0.001).

* *r*>0.30 after adjustment for age.

† *p*<0.05 for sex difference (Fisher r-to-z transformation).

ATR, anterior thalamic radiation; BDR, Buschke delayed recall; CC, corpus callosum; CST, corticospinal tract; HC, hippocampus; HI, hindered isotropic; IF, isotropic free water; IFO, inferior fronto-occipital; IFSF, inferior frontal superior frontal; ILF, inferior longitudinal fasciculus; LMD, logical memory delayed recall; ND, neurite density; RI, restricted isotropic; SCS, superior corticostriatal; SIF, striatal inferior frontal; SLF, superior longitudinal fasciculus; VRD, visual reproduction delayed recall.

### Associations between microstructure and cognitive function, adjusted for age

**Figure 3:**
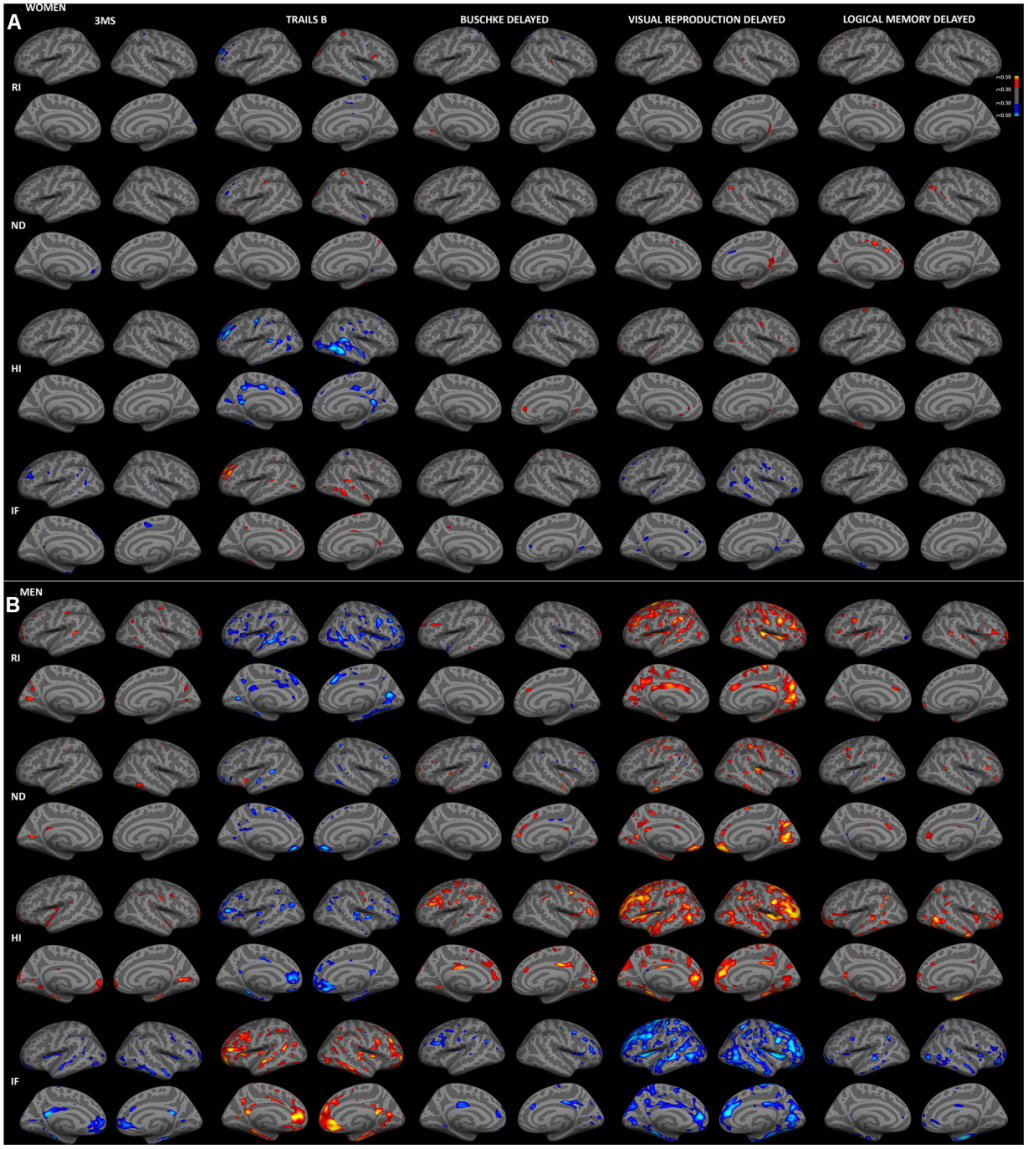
**Sex-stratified associations between cortical gray matter microstructure and cognitive function, adjusted for age.** Partial correlations between RSI metrics and cognitive test scores, adjusted for age and education, are shown for women (**A**) and men (**B**) (*r*>0.30). Figure conventions are the same as for Figure 1.

We previously reported stronger correlations between RSI measures and age for women than men in this cohort [27]. Thus, to examine whether sex differences in RSI-cognition correlations were attributable to differences in correlations with age, regressions were repeated with adjustment for age. As illustrated in Figure 3, correlations between cortical gray matter microstructure and cognitive function were substantially attenuated when age was included in regression models. For women, moderate (*r*>0.30) correlations remained between Trails B and HI in temporal, rostral middle frontal and cingulate regions, but correlations for other measures were minimal after age adjustment. Correlations among men were less attenuated by age adjustment. Widespread associations persisted for isotropic diffusion (RI, HI, IF) with Trails B and visual recall, with the strongest foci in medial and dorsolateral prefrontal, and medial parieto-occipital cortices. More limited associations were present for 3MS, Buschke recall and logical memory.

As shown in Table 2, correlations of hippocampal and fiber microstructure with cognitive function were also attenuated by adjustment for age. For women, correlations were strongly attenuated, with moderate correlations (*r*>0.30) remaining for hippocampus, forceps minor and corpus major, whereas for men moderate correlations persisted in a range of regions.

### Mediation of age effects on cognitive function by brain microstructure

**Figure 4:**
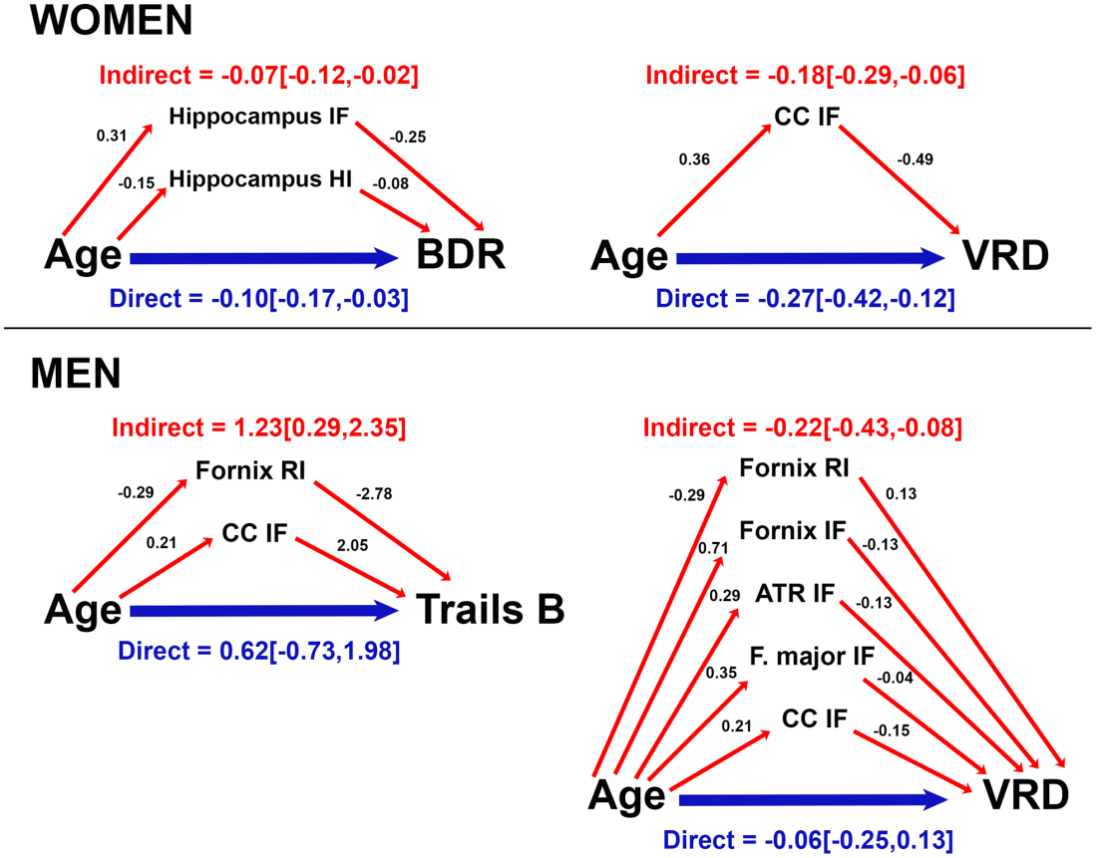
**Mediation of age effects on cognitive function by microstructure for women and men.** Direct (blue) and indirect (red) effects of age (coefficient [95% confidence interval]) are shown for cognitive scores mediated by microstructure. Coefficients for effects of age on RSI metrics, and effects of RSI metrics on cognitive score are provided. For ease of interpretation, RSI scores were converted to a similar order of magnitude as age and cognitive scores by multiplying raw RSI scores (range 0-1) by 100 (converted range 0-100). Coefficients are unstandardized and reflect the magnitude of change in RSI metric (or cognitive score) per year of age (or 1/100 unit increase in RSI metric). Data are shown for models in which total and indirect effects are significant at *p*<0.05. (ATR, anterior thalamic radiation; BDR, Buschke delayed recall; CC, corpus callosum; F major, forceps major; IF, isotropic free water; HI, hindered isotropic; RI, restricted isotropic; VRD, visual reproduction delayed recall.

Because of the robust correlations of age with microstructure and cognitive function, and between microstructure and cognitive function, we conducted mediation analyses to evaluate whether RSI mediated effects of age on cognition, and whether these mediators differed by sex. RSI metrics and cognitive scores that correlated with one another and with age were assessed as candidate RSI-cognition mediator pairs. Direct (age on cognition), and indirect (age on cognition via RSI) effects for models in which RSI mediated age-related cognitive function (i.e., significant indirect effect) for women and men are presented in Figure 4. For both sexes, corpus callosum IF mediated effects of age on visual recall. For women, hippocampal isotropic diffusion additionally mediated age effects on Buschke recall. For men, fornix RI and corpus callosum IF also mediated age effects on Trails B, and fornix, ATR, and forceps major isotropic diffusion mediated age effects on visual recall. Cortical gray matter RSI did not meet criteria for mediation.

## DISCUSSION

In this study, we observed that restricted, hindered and free water diffusion in gray and white matter were strongly associated with performance in multiple cognitive domains in community-dwelling older adults. Associations between microstructural compromise and cognitive deficits were largely attributable to advancing age, particularly for women, though select correlations between microstructure and cognitive performance were not fully explained by variability in age. Isotropic diffusion mediated age-related cognitive variability for both men and women, but mediation patterns were sex-specific. Hippocampal and corpus callosum microstructure mediated verbal and visuospatial memory for women, and fiber microstructure mediated executive function and visuospatial memory for men.

Microstructure strongly correlated with executive function and visual memory, and more weakly with episodic memory. Executive functions and processing speed demonstrate pronounced decline in typical aging [30], while episodic memory deficits manifest in both normal and pathological aging [31]. The more limited studies of visuospatial memory suggest that impairments in normal aging may reflect aggregate dysfunction in visuospatial processing, attention, working memory or contextual memory [32]. The cytoarchitectural markers of cognitive aging identified here thus align with phenotypes expected for typical aging, and may be beneficial in distinguishing patterns of normal brain aging from pathogenic changes.

Others have also reported correlations of neurite density with executive function and learning [18], and that orientation dispersion mediates effects of age on executive function [19], with predominantly frontally localized associations. Though we also observed strong associations with dorsolateral and medial prefrontal cortex isotropic diffusion, in line with a crucial role for the prefrontal cortex in supporting executive functions [33], widespread correlations were also present throughout the hippocampus, cortex and white matter skeleton (including foci in prefrontal, parietal, and medial and superior temporal cortices, along with fornix, anterior thalamic radiation, and commissural fibers). Our results expand upon prior observations that frontal regions are highly vulnerable to age-related injury [34] but perhaps not selectively [35], to suggest that extended network disruption may account for the breadth of cognitive deficits that emerge in later years. Although the logical memory and word list tests both probe verbal memory, distinct topographic associations were observed for these measures. This finding is consistent with evidence that they tap into meaningfully distinct cognitive functions subserved by unique neural circuitry, as list learning relies more heavily on executive processing [36] and is more sensitive to mild cognitive impairment [37] than logical memory. Particularly for women, microstructural correlates of word list recall mapped with regions of the default network, known to support episodic memory [38]. Logical memory performance was associated with a notable medial temporal cluster of isotropic diffusion. Medial temporal atrophy correlates with logical memory performance strongly in Alzheimer’s disease but minimally in normal aging [39], suggesting that RSI may be more sensitive to subtle cellular changes associated with logical memory decline in the absence of neurodegeneration. Widespread associations were present for visual memory, perhaps reflecting the diverse cognitive functions required for visual recall [32], including occipitoparietal foci that were absent for other cognitive scores and may relate to visuospatial components of this task.

We previously identified tighter correlations of age with cortical and fiber microstructure for women than for men of this cohort [27], and here extend this finding to report that microstructure better predicts cognitive performance in women. However, this difference was strongly attenuated by adjustment for age, and microstructure mediated effects of age on cognitive performance for both sexes. Thus, among our sample of older adults spanning the age range of 56-99 years, advancing age may elicit greater risk for microscopic brain injury in women, though cognitive repercussions of cytoarchitectural damage appear consequential for both sexes. Microstructural changes may begin over a decade earlier in men than women [40], accompanied by an earlier shift of functional decline in men [41]. According to this scenario, women in our sample may have experienced steeper age-related microstructural change than men, who had undergone comparable decline at an earlier age. Indeed, men had lower hindered and higher free diffusion in gray matter, perhaps indicating longer-standing neural injury than for women within the same age range. Distinct ages of onset of microstructural injury for men and women could account for prior inconclusive reports of sex differences in microstructural brain aging [23–25]. Examining men and women together could potentially mask associations between microstructure and cognition that differ by sex, possibly explaining the relatively more limited correlations reported in prior advanced diffusion MRI studies than observed here [18, 19]. Longitudinal studies of microstructural change in aging men and women will help to address these issues.

Mediation patterns differed by sex in both microstructural mediators and cognitive targets. For women, hippocampal isotropic diffusion mediated age effects on verbal memory, a task in which they demonstrated superior performance than men. For men, fiber isotropic diffusion mediated age effects on visuospatial memory and executive function. Relative to women, men perform better on visuospatial memory tasks [42], and in this cohort have demonstrated slower executive function decline [43]. These findings imply that neural microstructure closely mediates declines in functions that have been well preserved, and thus may support maintenance of cognitive abilities with age. Our data corroborate reports that hippocampal and white matter microstructure mediate effects of age on executive and visuospatial abilities [12, 17, 19]. Whereas others identified no microstructural mediators of age-related differences in verbal memory [14–16], here hippocampal isotropic diffusion mediated age-related variability in verbal memory for women, perhaps reflecting the more comprehensive characterization of tissue cytoarchitecture by RSI, cohort differences, or the sex-specificity of anatomical mediators of cognitive aging. Further investigation is needed to characterize neurobiological, hormonal or behavioral factors underlying differential rates of brain and cognitive aging for men and women.

Isotropic measures across diffusion scales most robustly correlated with cognition and mediated age-related cognitive variability, with weaker effects for ND. Spherical compartments such as cell bodies, synapses or extracellular space would be conducive to restricted or hindered isotropic diffusion, whereas CSF would permit free diffusion. Considering that relatively minimal neuronal death occurs during normal aging [1], redistribution of restricted and hindered fractions to the free fraction may more likely reflect cell shrinkage or dystrophy, loss of neurites or synapses, or demyelination [44, 45], than cell death. Supporting this interpretation, correlations were robust to adjustment for cortical thickness, suggesting that the differences in diffusion properties observed here reflect morphometric cellular changes independent of atrophy. Glial activation [46] or inflammation [47] may also contribute to functional disturbances with age, and can be captured by multi-compartment diffusion MRI parameters [48].

The RBS cohort is homogeneous in terms of race, education and socioeconomic class, which limits generalizability but also minimizes confounding by these factors. The cross-sectional design may introduce selection bias and precludes identifying patterns of microstructural change that predict cognitive trajectories. Because neuropathological data were unavailable, we were unable to exclude the possibility that preclinical neurodegenerative disease influenced brain or cognitive measures. However, when data from three individuals with poor global cognitive performance (3MS<80) [49] were removed, results were essentially unchanged. Finally, because RSI indirectly assesses tissue architecture, we cannot definitively identify cellular properties underlying differences in diffusion metrics.

## CONCLUSIONS

This study observed robust associations between gray and white matter microstructure and cognitive function in a community-dwelling sample of older adults. Whereas age may more strongly elevate risk for microstructural compromise and cognitive decline in women, cytoarchitectural injury appears to mediate cognitive decline with age for both sexes. The sex-specific patterns by which microstructure mediates age-related cognitive deficits suggest that men and women follow distinct trajectories of brain aging with divergent cognitive consequences. RSI may provide sensitive biomarkers of incipient cognitive decline to aid in monitoring interventions tailored to men and women intended to preserve brain health in late life.

## MATERIALS AND METHODS

### Participants

Participants of the RBS, a longitudinal study of community-dwelling older adults in southern California, who attended a research visit from 2014-2016 were eligible for study inclusion. Exclusion criteria included history of head injury, stroke, neurological disease, treatment for an alcohol use disorder, or safety contraindication for MRI. Of those who completed MRI and neuropsychological testing (*N*=154), six were excluded due to poor data quality and one due to severe white matter disease, leaving a final sample of 147 participants.

Study procedures were approved by the University of California, San Diego Human Research Protections Program Board and participants provided informed written consent prior to participation.

### Cognitive assessment

The neuropsychological test battery was administered by a trained examiner in a quiet room on the same day as the imaging session. The 3MS is a cognitive screening tool that assesses multiple cognitive domains. Trails B assesses executive function and processing speed, measuring time to completion to connect a sequence of alternating numbers letters and numbers with faster times indicating better performance. The Buschke Selective Reminding Test measures verbal episodic memory; total recall and 20-minute delayed recall were analyzed here. The Wechsler Visual Reproduction test evaluates visual memory of a series of five designs, immediately and after a 20-minute delay. The Wechsler Logical Memory subtest, story A, required participants to recall a story that was verbally accounted, either immediately or after a 20-minute delay.

### Health and lifestyle assessment

Education level was acquired at enrollment and converted to years of education. Height and weight were measured with the participant wearing light clothing and without shoes; BMI was used as an estimate of obesity. Blood pressure was measured in seated, resting participants by a trained nurse and the mean of two readings, taken five minutes apart, was used for analysis. Participants were considered hypertensive if they had an average systolic blood pressure reading >140, diastolic blood pressure reading >90, were taking antihypertensive medication, or reported a physician diagnosis of hypertension. Information on smoking (never versus former; there were no current smokers), exercise (three or more times per week, yes/no), alcohol consumption (non-drinker/drinker), and history of diabetes was obtained from standard questionnaires.

### Imaging data acquisition

MRI data were acquired on a 3.0 Tesla Discovery 750 scanner (GE Healthcare, Milwaukee, WI, USA) with an eight-channel phased array head coil at the UC San Diego Center for Functional MRI. The MRI sequence included a three-plane localizer; a sagittal 3D fast spoiled gradient echo T_1_ -weighted volume optimized for maximum gray/white matter contrast (TE=3.2 ms, TR=8.1 ms, inversion time=600 ms, flip angle=8°, FOV=256×256 mm, matrix=256×192, slice thickness=1.2 mm, resampled to a resolution of 1×1×1.2 mm, scan time 8:27); and an axial 2D single-shot pulsed-field gradient spin-echo echo-planar diffusion-weighted sequence (45-directions, b-values=0, 500, 1500, 4000 s/mm2, one b=0 volume and 15 gradient directions for each non-zero b-value; TE=80.6 ms, TR=7 s, FOV=240×240 mm, matrix=96×96, slice thickness=2.5 mm, resampled to a resolution of 1.875×1.875×2.5 mm, scan time 6:34). An additional b=0 volume was collected prior to the diffusion sequence with reverse phase-encode polarity for B_0_ distortion correction.

### Data processing

Image data processing integrated FreeSurfer (http://surfer.nmr.mgh.harvard.edu) with tools developed in-house, as previously described [50]. Briefly, RSI data were corrected for motion, eddy current, B_0_ susceptibility, and gradient nonlinearity distortions [51–53], and images containing uncorrectable artifacts were excluded. Gray matter, white matter, and CSF boundaries were identified on T_1_ -weighted structural images using FreeSurfer’s automated cortical reconstruction, and cortical thickness was computed as the distance between the white matter and pial surfaces [54]. After coarse pre-alignment to atlas images, RSI volumes were registered to T_1_ data using mutual information [55]. Data were then resampled to the original RSI acquisition resolution using cubic interpolation, and a registration matrix was created to specify the rigid-body transformation between RSI and T_1_-weighted images.

RSI metrics included RI, ND, HI, and IF. RI is computed as the 0^th^ spherical harmonic of the restricted compartment and measures intracellular integrity. ND, which combines the 2^nd^ and 4^th^ spherical of the restricted compartment, reflects oriented intracellular diffusion while accounting for crossing fibers, and thus likely reflects the fraction of axons and dendrites. HI is computed as the 0^th^ spherical harmonic of the hindered compartment, thought to reflect large cell bodies or the extracellular space. IF estimates the CSF fraction. Cortical gray matter RSI metrics were computed from 0.8-2.0 mm from the gray/white matter boundary normal to the cortical ribbon. RSI maps were projected onto the cortical surface and registered to common space (*fsaverage)* and smoothed with a FWHM 10 mm kernel. RSI measures were computed in the hippocampus, automatically segmented according to a subcortical atlas [56], and in 33 gray matter ROIs labeled according to the Desikan-Kiliany atlas [57]. HI was not examined in fiber tracts because white matter architecture is poorly characterized by the hindered fraction [26], but all other RSI metrics were computed in fifteen white matter fiber tracts labeled using a probabilistic atlas (AtlasTrack) [58]. Voxels containing primarily gray matter or CSF were excluded from white matter tracts [56]. Global RSI measures were computed as the average across all cortical gray matter or across all fiber tracts.

### Statistical analysis

Age, health and lifestyle variables, and global RSI measures were compared between men and women using independent-samples t-tests (continuous variables) or chi-squared tests (categorical variables). Cognitive test scores, corrected for education, were compared between sexes using univariate ANOVA. Partial correlations between age and cognition, adjusted for education, were computed for women and men, and correlations were compared between sexes using Fisher r-to-z transformation.

To examine associations between whole-brain microstructure and cognitive function, sex-stratified partial correlations were computed between each global RSI metric and each cognitive measure. Sensitivity analyses included marital status (married/unmarried) and residential status (living alone/cohabitating) as additional covariates in partial correlations between global RSI metrics and cognitive measures.

To examine the topographic distribution of correlations between microstructure and cognition, sex-stratified general linear models (GLM) for each cognitive measure were performed on gray matter RSI surface maps. Sex-stratified partial correlations were repeated for cortical gray matter ROIs demonstrating moderate correlations (*r*>0.30) on RSI surface maps for either sex, as well as for RSI metrics in hippocampus and fiber tracts. Partial correlations and GLMs between RSI and cognitive scores were adjusted for education. Sex differences in correlations were assessed using Fisher r-to-z transformation.

To compare RSI-cognition associations with associations for cortical thickness, sex-stratified GLMs were computed on cortical thickness maps for each cognitive score, adjusted for education. To evaluate whether associations between microstructure and cognitive function were attributable to atrophy, GLMs for each cognitive and RSI measure were computed across all subjects, adjusted for sex and education, and with vertex-wise correction for cortical thickness.

To determine whether associations between microstructure and cognitive function were attributable to age, sex-stratified GLMs and partial correlations were conducted with age as an additional covariate.

Mediation of age effects on cognitive performance by microstructure was examined separately for women and men. Candidate measures included RSI metrics in fiber tracts, cortical gray matter ROIs, or hippocampus demonstrating significant correlations with age and with cognitive function (adjusted for age and education), and cognitive tests scores that correlated with age (adjusted for education). For cognitive and RSI measures meeting these criteria, mediation analysis was performed using ordinary least squares path analysis using the PROCESS macro (model 4) in SPSS [59], with age as the independent variable, cognitive measure as the dependent variable, RSI metric as mediator, and education as a covariate. Significance of coefficients for direct (age directly on cognition), and indirect (age on cognition via RSI) effects was assessed at a 95% confidence interval. For each cognitive score demonstrating mediation by RSI, mediation analysis was performed combining all significant RSI mediators into a single model.

Correlation coefficients of 0.30 or greater were considered medium (*p*<0.004 for women; *p*<0.02 for men) and correlation coefficients of 0.50 or greater (*p*<0.001) were considered strong. For ROI analyses, significance was set to *p*<0.05 and adjusted for multiple comparisons across 15 fiber tracts (*p*<0.003) or 33 gray matter ROIs (*p*<0.0015). Fisher tests were evaluated at *p*<0.05. For vertex-wise cortical gray matter GLMs, thresholds were set to medium or greater correlations (*r*>0.30). Vertex-wise Fisher tests and RSI GLMs with vertex-wise adjustment for thickness were examined at *p*<0.05, FDR corrected for multiple comparisons, and Fisher tests were additionally examined at an uncorrected threshold of *p*<0.01.

Analyses were performed in Freesurfer version 5.3.0 and SPSS version 26.0 (IBM Corp, Armonk, NY, USA).

## Supplementary Material

Supplementary Figures

Supplementary Tables
