# Supplementary material for: Brain microstructure mediates sex-specific patterns of cognitive aging
Source: Aging (Albany NY). 2021 Jan 28;13(3):3218–38. doi: 10.18632/aging.202561 (PMC7906181; doi:10.18632/aging.202561)
Supplement: Supplementary Figures [file aging-13-202561-s001.pdf]

SUPPLEMENTARY FIGURES

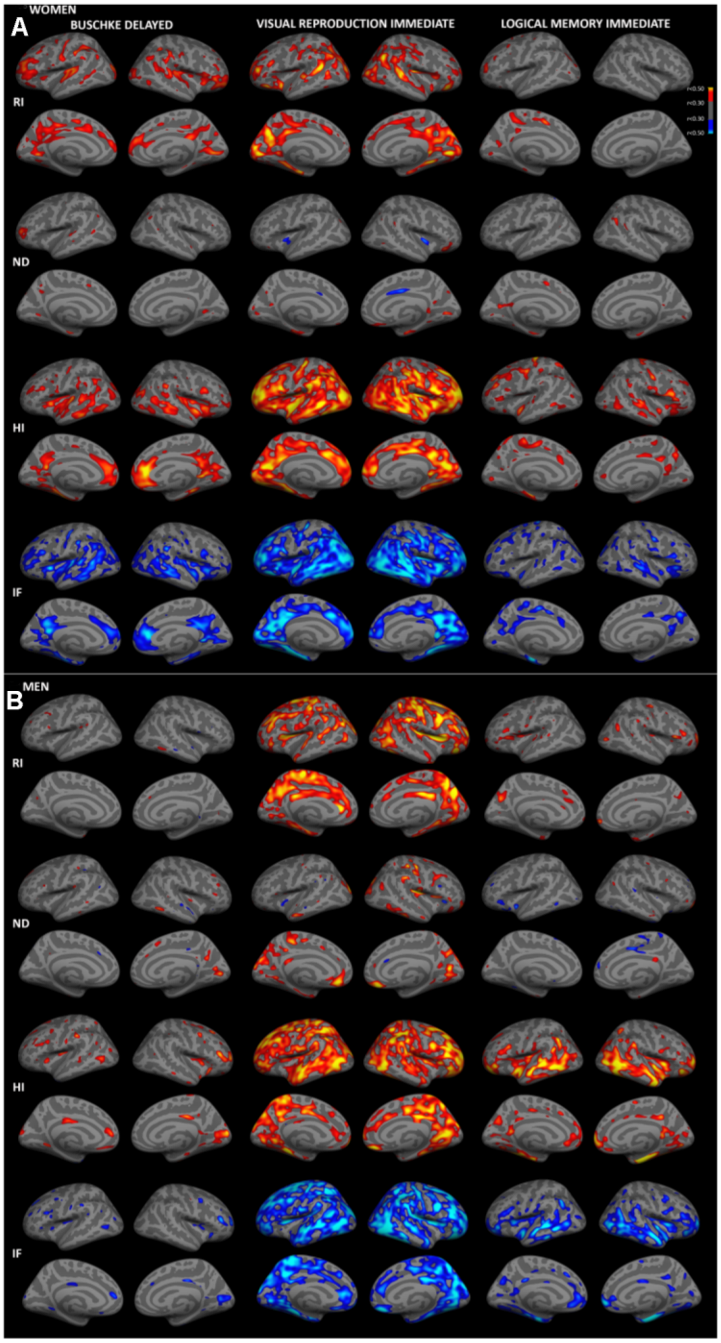

Supplementary Figure 1. Associations between cortical gray matter microstructure and immediate recall. Partial correlations between RSI metrics and immediate recall scores, adjusted for education, are shown for women (**A**) and men (**B**). Warm colors indicate positive correlations and cool colors indicate negative correlations (*r*>0.30). (HI, hindered isotropic; IF, isotropic free water; ND, neurite density; RI, restricted isotropic).

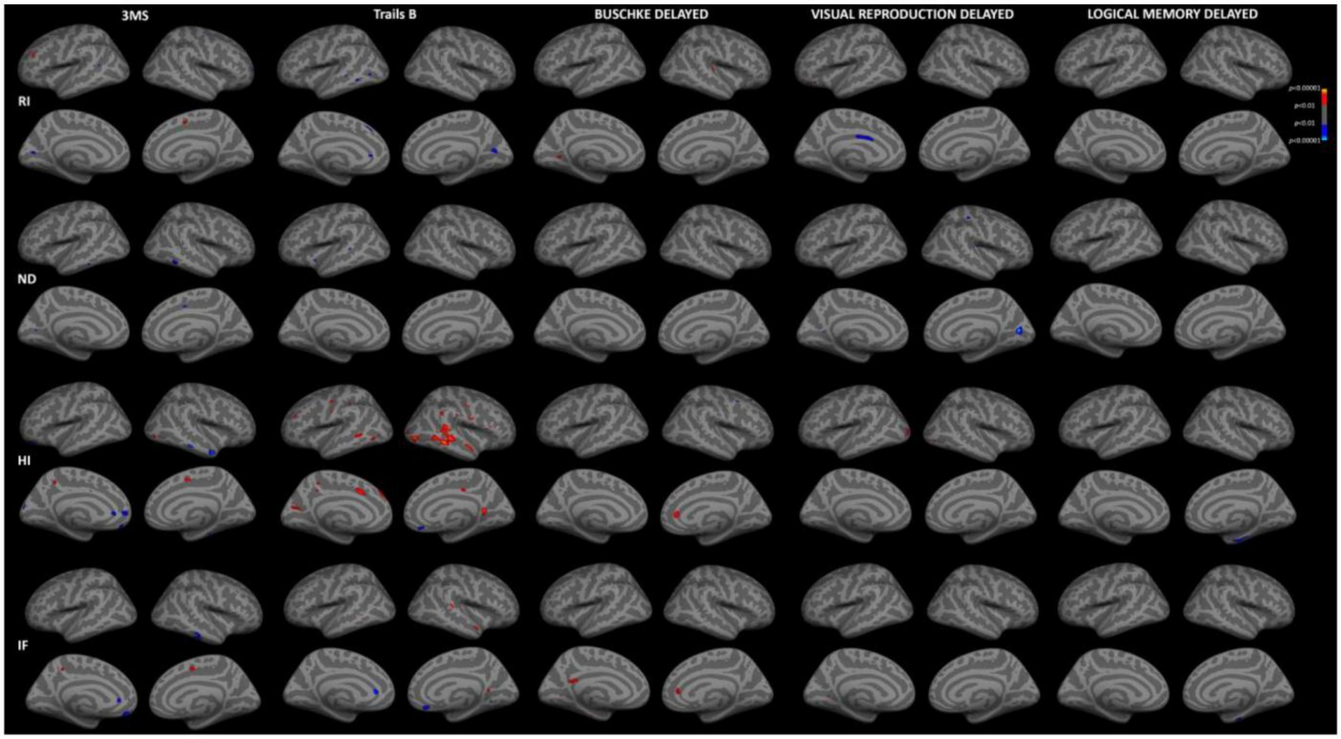

Supplementary Figure 2. Sex differences in correlations between cortical gray matter microstructure and cognitive function. Correlations between RSI metrics and cognitive test scores for men and women were compared using Fisher r-to-z transformation. Warm colors show regions with stronger correlations for women and cool colors show regions with stronger correlations for men (*p*<0.01, uncorrected). (HI, hindered isotropic; IF, isotropic free water; ND, neurite density; RI, restricted isotropic).

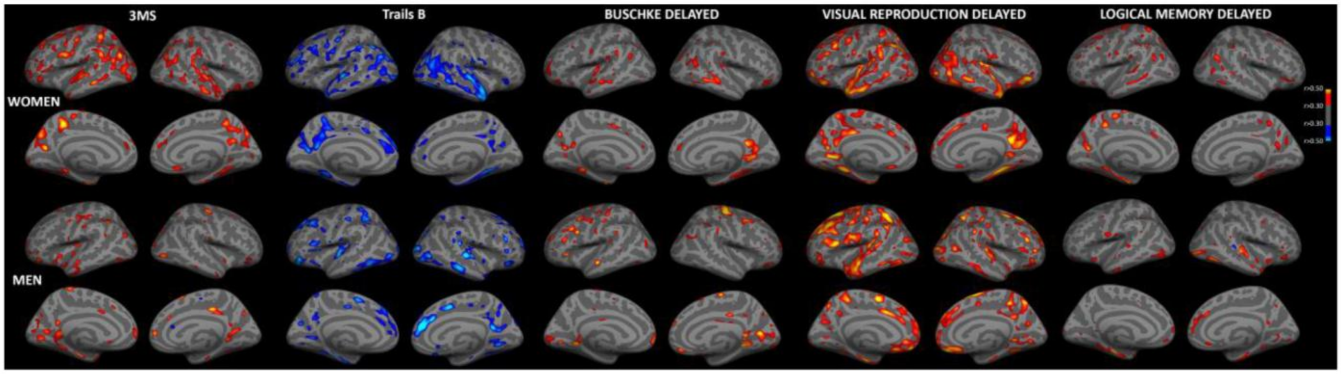

Supplementary Figure 3. Associations between cortical thickness and cognitive function. Partial correlations between cortical thickness and cognitive test scores, adjusted for education, are shown for women (**A**) and men (**B**). Warm colors indicate positive correlations and cool colors indicate negative correlations (*r*>0.30). For Trails B, lower scores indicate better performance.

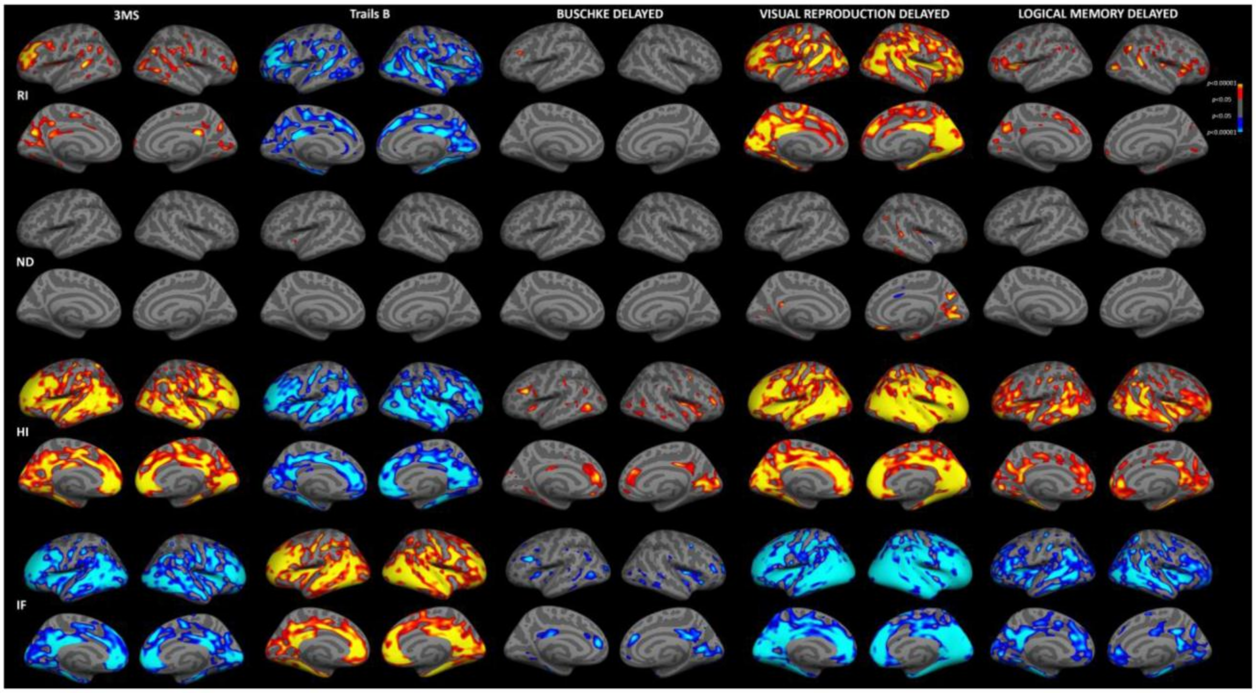

Supplementary Figure 4. Associations between cortical gray matter microstructure and cognitive function, adjusted for thickness. Partial correlations between RSI metrics and cognitive test scores, adjusted for sex, education and cortical thickness, are shown for all subjects. Warm colors indicate positive correlations and cool colors indicate negative correlations (p<0.05, FDR corrected).
