# Supplementary material for: Brain microstructure mediates sex-specific patterns of cognitive aging
Source: Aging (Albany NY). 2021 Jan 28;13(3):3218–38. doi: 10.18632/aging.202561 (PMC7906181; doi:10.18632/aging.202561)
Supplement: Supplementary Tables [file aging-13-202561-s002.pdf]

SUPPLEMENTARY TABLES

Supplementary Table 1. Correlations between age and cognitive function (partial *r*, adjusted for education), between age and global RSI metrics (Pearson's correlation), and between global RSI metrics and cognitive test scores (partial *r*, adjusted for education), stratified by sex.

		Women						Men					
		**Age**	**3MS**	**Trails B**	**BDR**	**VRD**	**LMD**	**Age**	**3MS**	**Trails B**	**BDR**	**VRD**	**LMD**
	Age	-	**-0.50**	**0.57**	**-0.52***	**-0.63**	-0.40	-	-0.21	0.37	-0.12	-0.41	-0.47
White matter fibers	RI	**-0.70***	0.30	-0.42	0.46*	**0.52**	0.33	-0.48	0.27	-0.47	-0.02	0.42	0.33
	ND	**-0.60***	0.32	-0.36	0.37*	**0.51**	0.35	-0.29	0.27	-0.37	-0.07	0.34	0.20
	IF	**0.72**	-0.47	0.49	-0.48*	**-0.60**	-0.36	**0.52**	-0.36	0.46	-0.06	**-0.56**	-0.45
Gray matter	RI	**-0.70**	0.30	-0.36	0.34	0.49	0.30	-0.37	0.28	-0.45	0.06	0.49	0.25
	ND	-0.16	0.16	-0.06	0.11	0.22	0.14	0.12	0.10	-0.28	0.09	0.38	0.09
	IF	**0.77***	-0.46	**0.51**	-0.35	**-0.59**	-0.35	0.46	-0.41	0.46	-0.20	**-0.60**	-0.38
	HI	**-0.72***	0.47	**-0.53**	0.31	**0.55**	0.34	-0.49	0.47	-0.39	0.27	**0.57**	0.38

Bold values indicate $r>0.50$.
*$p<0.05$ for sex difference (Fisher r-to-z transformation).
BDR, Buschke delayed recall; HI, hindered isotropic; IF, isotropic free water; LMD, logical memory delayed recall; ND, neurite density; RI, restricted isotropic; VRD, visual reproduction delayed recall.

Supplementary Table 2. Correlations between cognitive function and global RSI metrics (partial *r*), adjusted for education, marital status (unmarried/married), and living status (alone/cohabitating) for women.

		3MS	Trails B	BDR	VRD	LMD
White matter fibers	RI	0.18	-0.38	0.40	0.46	0.30
	ND	0.20	-0.28	0.29	0.46	0.32
	IF	-0.33	0.39	-0.40	**-0.54**	-0.31
Gray matter	RI	0.22	-0.33	0.29	0.45	0.27
	ND	0.10	0.01	0.06	0.19	0.11
	IF	-0.37	0.46	-0.27	**-0.55**	-0.31
	HI	0.37	-0.47	0.23	**0.51**	0.29

Bold values indicate $r>0.50$.
Analyses for men were not conducted due to the low number (5%) of men who were unmarried or living alone.
BDR, Buschke delayed recall; HI, hindered isotropic; IF, isotropic free water; LMD, logical memory delayed recall; ND, neurite density; RI, restricted isotropic; VRD, visual reproduction delayed recall.

Supplementary Table 3. Partial correlations (partial *r*) between hippocampal and fiber tract RSI metrics and cognitive test scores, for immediate recall measures.

Region	Measure	Women			Men		
		BTR	VRI	LMI	BTR	VRI	LMI
Hippocampus	RI	0.27	0.31	0.15	-0.06	0.18	0.09
	ND	0.31 †	0.43	0.24	-0.19	0.20	0.08
	IF	-0.44	-0.22	-0.31	-0.17	-0.25	**-0.56 ***
	HI	0.28	0.03	0.20	0.23	0.18	0.44
Fornix	RI	0.47 †	0.48	0.21	0.05	0.43 *	0.36
	ND	0.36 †	0.46	0.19	0.01	0.36	0.38
	IF	**-0.50** †	-0.47	-0.24	-0.11	-0.46 *	-0.44
Cingulum	RI	0.36 †	**0.50**	0.24	-0.09	0.35	0.26
	ND	0.17	0.03	0.20	0.04	0.33	0.05
	IF	-0.08	-0.14	-0.08	-0.10	-0.35	-0.46 * †
Parahippocampal Cingulum	RI	0.21	0.33	0.15	-0.05	0.21	0.07
	ND	0.10	0.24	0.11	-0.25 †	-0.02	-0.04
	IF	-0.18	-0.08	-0.06	-0.19	-0.34	-0.33
CST	RI	0.43 †	0.41	0.22	-0.06	0.27	0.10
	ND	0.08	0.26	0.16	-0.06	0.12	0.17
	IF	-0.34 †	-0.49	-0.23	0.05	-0.31	-0.30
ATR	RI	0.39 †	**0.52**	0.28	-0.10	0.30	0.25
	ND	0.37 †	0.41	0.25	-0.11	0.25	0.21
	IF	**-0.50** †	**-0.53**	-0.32	-0.15	-0.39	-0.34
Uncinate	RI	0.30 †	0.44	0.26	-0.14	0.23	0.15
	ND	0.26	0.32	0.27	0.04	0.24	0.09
	IF	-0.45 †	-0.39	-0.35	0.00	-0.26	-0.31
ILF	RI	0.31 †	0.38	0.22	-0.03	0.33	0.19
	ND	0.28	0.28	0.23	-0.01	0.22	0.15
	IF	-0.38 †	-0.42	-0.25	0.01	-0.40 *	-0.43 *
IFO	RI	0.37 †	0.47	0.28	-0.11	0.26	0.16
	ND	0.34	0.38	0.31	0.05	0.20	0.23
	IF	-0.44 †	**-0.53**	-0.26	-0.10	-0.38	-0.39
Forceps major	RI	0.35 †	0.48	0.20	0.00	0.27	0.18
	ND	0.35	0.38	0.30	0.18	0.48 *	0.27
	IF	-0.48 †	**-0.54**	-0.24	-0.11	-0.42	-0.40
Forceps minor	RI	0.42 †	0.41	0.32	-0.19	0.28	0.24
	ND	0.41 †	0.47	0.34	-0.16	0.29	0.23
	IF	-0.47 †	-0.40	-0.36	0.04	-0.26	-0.32
CC	RI	0.45 †	**0.51**	0.33	-0.02	0.40 *	0.24
	ND	0.42 †	**0.51**	0.35	0.08	0.43 *	0.25
	IF	-0.48 †	**-0.55**	-0.31	-0.06	-0.48 *	-0.33
SLF	RI	0.35	0.35	0.27	0.08	0.33	0.25
	ND	0.19	0.16	0.36	0.15	0.23	0.19
	IF	-0.25	-0.34	-0.26	-0.12	-0.39	-0.34
SCS	RI	0.42 †	0.44	0.30	0.09	0.34	0.20
	ND	0.04	0.21	0.20	0.02	-0.02	-0.07
	IF	-0.32	-0.47	-0.28	-0.02	-0.30	-0.17
SIFC	RI	0.39 †	**0.53**	0.34	-0.10	0.34	0.28
	ND	0.21	0.26	0.30 †	-0.01	-0.04	-0.06
	IF	-0.36	-0.44	-0.32	-0.08	-0.19	-0.21
IFSFC	RI	0.40 †	0.40	0.30	0.06	0.46 *	0.26
	ND	0.31	0.34	0.31	0.09	0.23	0.16
	IF	-0.32	-0.41	-0.27	-0.05	-0.33	-0.23

Bold values indicate r>0.50 (p<0.001).

* r>0.30 after adjustment for age.

† p<0.05 for sex difference (Fisher r-to-z transformation).

ATR, anterior thalamic radiation; BTR, Buschke total recall; CC, corpus callosum; CST, corticospinal tract; HC, hippocampus; HI, hindered isotropic; IF, isotropic free water; IFO, inferior fronto-occipital; IFSF, inferior frontal superior frontal; ILF, inferior longitudinal fasciculus; LMI, logical memory immediate recall; ND, neurite density; RI, restricted isotropic; SCS, superior corticostriatal; SIF, striatal inferior frontal; SLF, superior longitudinal fasciculus; VRI, visual reproduction immediate recall.
